# Decision-Making, Pro-variance Biases and Mood-Related Traits

**DOI:** 10.5334/cpsy.114

**Published:** 2024-08-21

**Authors:** Wanjun Lin, Raymond J. Dolan

**Affiliations:** 1 Max Planck University College London Centre for Computational Psychiatry and Ageing Research, University College London, London WC1B 5EH, UK; 2 Welcome Centre for Human Neuroimaging, University College London, London WC1N 3BG, UK

**Keywords:** Risk-seeking, Rumination, Uncertainty, Pro-variance bias, Bayesian, Conditional Value-at-Risk

## Abstract

In value-based decision-making there is wide behavioural variability in how individuals respond to uncertainty. Maladaptive responses to uncertainty have been linked to a vulnerability to mental illness, for example, between risk aversion and affective disorders. Here, we examine individual differences in risk sensitivity when subjects confront options drawn from different value distributions, where these embody the same or different means and variances. In simulations, we show that a model that learns a distribution using Bayes’ rule and reads out different parts of the distribution under the influence of a risk-sensitive parameter (Conditional Value at Risk, CVaR) predicts how likely an agent is to prefer a broader over a narrow distribution (pro-variance bias/risk-seeking) under the same overall means. Using empirical data, we show that CVaR estimates correlate with participants’ pro-variance biases better than a range of alternative parameters derived from other models. Importantly, across two independent samples, CVaR estimates and participants’ pro-variance bias negatively correlated with trait rumination, a common trait in depression and anxiety. We conclude that a Bayesian-CVaR model captures individual differences in sensitivity to variance in value distributions and task-independent trait dispositions linked to affective disorders.

## Introduction

Value learning is fundamental to survival and well-being, with maladaptive responses manifesting in excessive risk avoidance linked to anxiety or depression (Lorian, Mahoney et al. 2012). A critical aspect of value learning relates to how agents deal with uncertainty (Dayan and Jyu 2003). For example, Tsetsos et al. (Tsetsos, Chater et al. 2012) showed that when participants are presented with two streams of numerical values drawn from two distributions with the same overall mean value but different variances, they show a preference for the broader over the narrower distribution, a finding also reported in macaque monkeys (Cavanagh, Lam et al. 2020). This disposition to prefer a broader over a narrow distribution, even when the two distributions have the same means, is referred to as a “pro-variance bias” (Cavanagh, Lam et al. 2020). However, while it is known that variances induce systematic choice biases, the precise cognitive mechanism leading to these biases is unclear.

Moeller et al. (Moeller, Grohn et al. 2021), in a trial-by-trial decision task where participants choose between pairs of options which varied both in terms of mean (high or low) and variance (broad or narrow), showed that a pro-variance bias emerges over the course of learning. This time-dependent emergence of a pro-variance bias was particularly marked for a set of options with higher-than-average means (both-high), with an opposite pattern seen when options had lower-than-average means. Notably, in these data, there were substantial individual difference effects in pro-variance biases where, for example, in the both-high condition, the percentages choosing the broader option ranged from about 90% to about 30%. However, here the individual differences in pro-variance bias might be confounded by direct exploration (sampling the uncertain option to reduce uncertainty (Wilson, Geana et al. 2014)). For example, Moeller et al. (Moeller, Grohn et al. 2021) used a learning paradigm in the context of a partial feedback design, wherein participants were provided with feedback solely on a chosen option. In the current study, we opted for a much-simplified task design involving complete feedback, a manipulation known to mitigate exploration (Findling, Skvortsova et al. 2019, Jahn, Grohn et al. 2023). This enables an examination of individual differences in pro-variance bias that closely accords with previous studies (Tsetsos, Chater et al. 2012, Cavanagh, Lam et al. 2020).

A candidate source of individual differences in pro-variance bias is an asymmetry in learning from positive and negative prediction errors, as captured in a 2-learning-rate Rescorla–Wagner model (2lr-RW) (Cazé and van der Meer 2013). An agent with a higher positive than negative learning rate (positive biased) would integrate more variances thereby increasing expectations, forming higher expectations with higher variances and becoming pro-variance biased, and vice versa for agents with negative biased learning rates. Consistent with this hypothesis, rodent data has shown that a negative learning bias measured in one task is associated with individual differences in risky choices, manifest in an entirely separate task as a preference for an option with a more variable amount of reward (Shabel, Murphy et al. 2014). Notably, the 2lr-RW model was the best-fitted model in the above-mentioned study (Moeller, Grohn et al. 2021), suggesting this class of model can capture the evolution of variance-induced biases.

A recent theoretical account (Gagne and Dayan 2022) has proposed a risk-sensitive parameter, Conditional Value at Risk (CVaR), as a novel way of modelling individual differences in risk sensitivity. CVaR can be defined as an outcome expectation for either a lower or upper part of a distribution (Filippi, Guastaroba et al. 2020). Within this framework, risk aversion originates from a negative read-out of possible outcomes, i.e. lower tail of a distribution (Gagne and Dayan 2022). The aforementioned models, namely an asymmetric learning bias and partial read-out of possible outcomes, provide competing hypotheses for risk-sensitive behaviours. In this study, using both simulation and empirical data we ask whether key parameters (relative positivity in learning rates and CVaR) used in such computational models relate to individual differences in pro-variance bias.

A wider relevance of decision-making under uncertainty is that increased risk aversion has been linked to anxiety and depression across a range of contexts, including decision questionnaires (Eisenberg, Baron et al. 1998), the Balloon Analogue Risk Task (Maner, Richey et al. 2007; Hevey, Thomas et al. 2017; Follett, Hitchcock et al. 2023) and probabilistic gambling tasks (Chandler, Wakeley et al. 2009; Charpentier, Aylward et al. 2017). Moreover, in depressed patients, high risk aversion is linked to poorer life satisfaction (Young, Goodmann et al. 2023) and a disposition to suicide (Baek, Kwon et al. 2017). These associations suggest that risk-related individual differences may serve as a potential vulnerability marker for mood disorders. Risk aversion is generally defined as a disposition to prefer an option with lower uncertainty/variance (Werner 2016). A pro-variance bias is a form of risk-seeking (the opposite of risk aversion). This leads us to examine for an association between the pro-variance bias and task-independent individual trait differences that relate to depression and anxiety.

**Figure 1:**
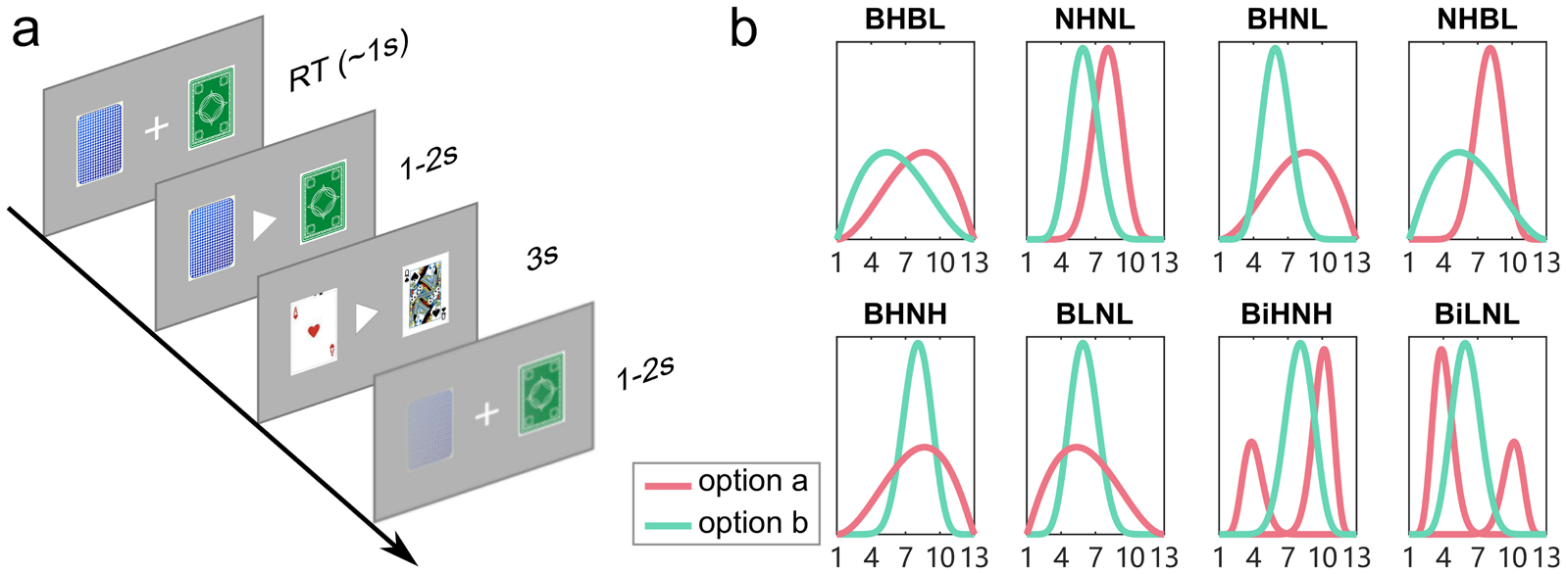
**Experimental design. a)** task structure. Within a task block, participants were presented with the same two card decks for 30 trials. After entering a choice, a triangle is presented in the centre of the screen pointing to the chosen deck. Following this, a card from each deck was shown to the participants. **b)** Block types. The two card decks in a block could have the same or different means (high or low) and variances (narrow or broad). In total this entailed eight different blocks comprising broad-high vs broad-low (BHBL), narrow-high vs narrow-low (NHNL), broad-high vs narrow-low (BHNL), narrow-high vs broad-low (NHBL), broad-high vs narrow-high (BHNH), broad-low vs narrow-low (BLNL), bimodal-high vs narrow-high (BiHNH), and bimodal-low vs narrow-low (BiLNL). The two decks presented in the bottom four blocks have the same numerical means but different variances. To incentivize participants, they were instructed that the points they accumulated throughout the game would be converted into an actual monetary reward.

We conducted an online experiment where we probed how individual differences in risk sensitivity related to traits of anxiety and depression. To implement the experiment online, we developed a simple and intuitive magnitude learning task using poker cards, where we independently manipulated both variance and mean. Within the task, participants had to choose between card decks associated with different value distributions. Critically, in half of the blocks, participants were presented with options having the same means but different variances (the bottom four blocks shown in Figure 1b). This design feature enabled us to examine the effect of mean and variance on pro-variance biases, i.e., preference for the broader options in equal-mean blocks, as well as determine if computational parameters, particularly a positivity bias in learning rates in the 2lr-RW, and CVaR in a Bayesian CVaR model (see methods for model details), relate to individual differences in pro-variance bias in both simulations and empirical data. Our overarching hypothesis was that pro-variance bias and risk-sensitive parameters would be associated with traits linked to mood disorder.

## Results

We present findings from two independent online general population samples: a discovery (107 participants recruited) and a replication study (117 participants were recruited). Twenty-eight participants from the discovery sample and 26 from the replication sample were excluded from analyses based on pre-specified criteria (see Methods).

### Effect of mean and variance on pro-variance bias

**Figure 2:**
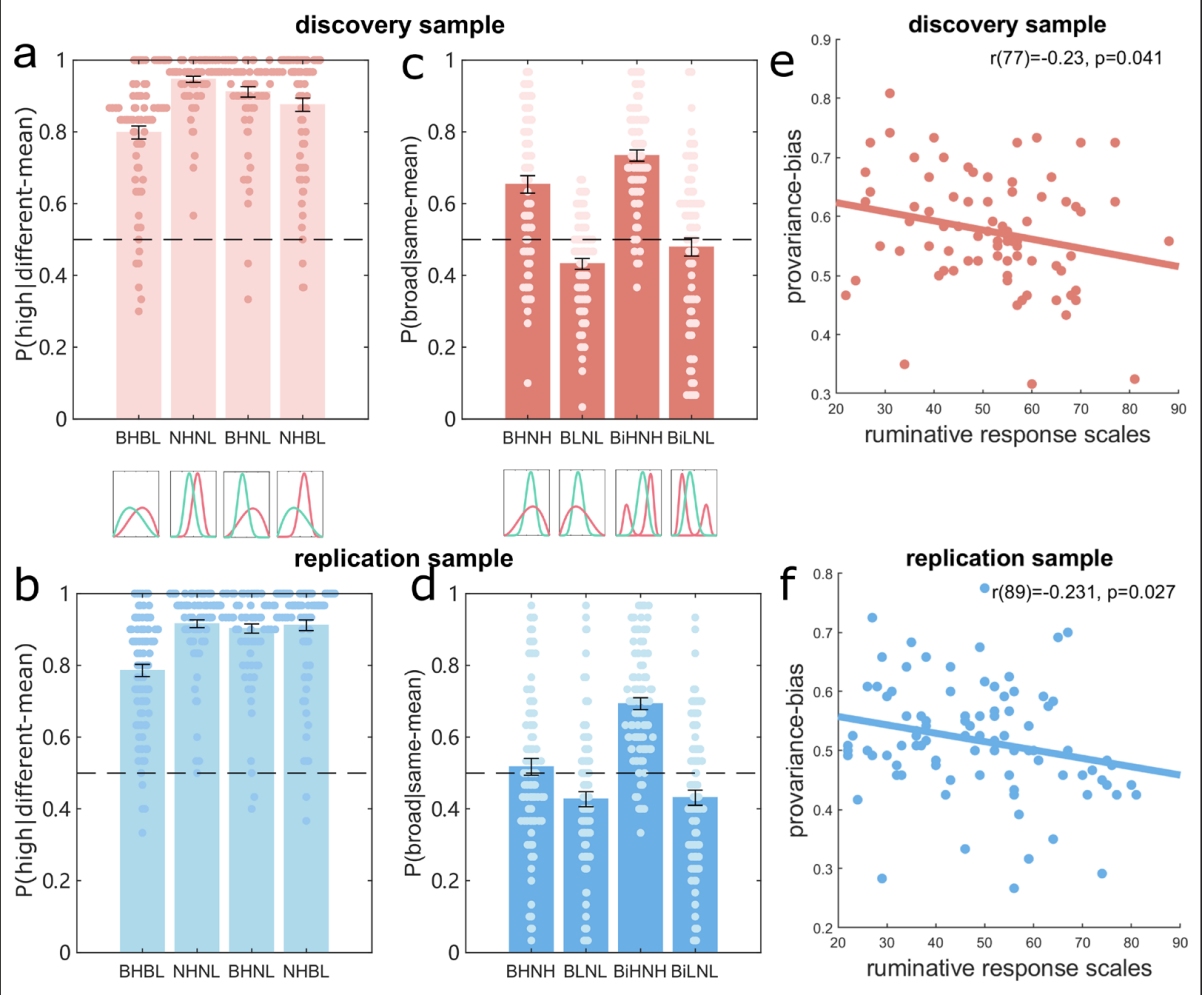
**Pro-variance bias and ruminations. a–b)** The percentages of choosing the option with a higher mean for the different-mean blocks for the discovery and replication samples, respectively. **c–d)** The percentages of choosing the option with a broader distribution for the same-mean blocks for the discovery and replication samples, respectively. The dots on each bar represent the data point for each participant for that block. **e–f)** the correlations between rumination scores and the mean pro-variance bias in the same-mean blocks for the discovery and replication samples, respectively. Error bars indicate standard errors (s.e.); *p < 0.05; **p < 0.01; ***p < 0.001.

First, we examined participants’ performances for the different-mean blocks (i.e., the top four blocks in Figure 1b, including the broad-high vs. broad-low (BHBL) block, the narrow-high vs narrow-low (NHNL) block, the broad-high vs narrow-low (BHNL) block, the narrow-high vs broad-low (NHBL) block). Participants chose the options with higher mean values (option a) (see Figure 2a&b) significantly above chance level (50%), in both the discovery (all t(78) > 16.45, p < .001) and replication samples (all t(90) > 16.90, p < .001). As expected, the percentage choosing the higher option in the BHBL block, where both options had broader distributions, was significantly lower than for the other three different-mean blocks (all t(78) >= –3.456, p <= .001 for the discovery sample, all t(90) <– 6.735, p < .001 for the replication samples.). This pattern showed that participants learned value differences well, and where options having broader distributions render accuracies in the BHBL block lower than is the case for other different-mean blocks.

Next, we examined for pro-variance biases by asking whether participants chose options with a broader distribution more in the same-mean blocks (the bottom four blocks in Figure 1b). For the discovery sample, participants chose the broader option significantly above chance level for the BHNH and BiHNH blocks (both t(78) > 6.34, p < .001), while choosing the narrow option significantly more for the BLNL block (t(78) = –4.466, p < 0.001) but not for the BiLNL block (t(78) = –.838, p = .405) (Figure 2c). In the replication sample, participants chose the narrower option significantly more in the BLNL and BiLNL block (both t(90) < –3.245, p ≤ 0.002), while choosing the broader option more in the BiHNH block (t(90) = 11.544, p < .001), but not for the BHNH block (t(90) = .729, p = .468) (Figure 2d). This response pattern suggests participants tend to be pro-variance biased in the both-high blocks but prefer the narrower option in the both-low mean blocks.

To formally examine the effect of mean and variance on pro-variance bias, we implemented a 2 × 2 (mean: high vs. low; variance: broad vs. bimodal) repeated ANOVA for the four same-mean blocks. This analysis revealed a significant main effect of mean (Figure 2c&d, discovery sample: F(1,78) = 118.836, p < .001; replication sample: F(1,90) = 73.384, p < .001). Post-hoc analyses indicated a significantly greater pro-variance bias in the BHNH block compared to the BLNL block (discovery sample: p < .001; replication sample: p = .019), and for the bimodal blocks (BiHNH > BiLNL both p < .001). Thus, we replicated the main behavioural results of Moeller et al.(Moeller, Grohn et al. 2021), who showed greater pro-variance bias for better-than-expected options, using a much-simplified task.

These analyses also revealed a significant main effect of variance (discovery sample: F(1,78) = 8.527, p = .005; replication sample: F(1,90) = 15.894, p < .001). However, an interaction of mean × variance was significant in the replication sample alone (F(1,90) = 19.120, p < .001), and not in the discovery sample (p = .323). Post-hoc analyses showed increased pro-variance bias in bimodal blocks for both-high mean blocks (p = .033 for the discovery sample, p < .001 for the replication sample) but not both-low mean blocks (both p > .507).

The results suggest that higher variances (bimodal distributions) further boost a pro-variance bias for options whose means are higher than expected. The observed pattern of results indicates participants were more risk-seeking when the environment yielded better-than-average outcomes and more risk-averse when the environment yielded worse-than-average outcomes. Higher variance environments served to enhance risk-seeking trends. Note, in our empirical data, we observed substantial individual differences in expression of a pro-variance bias, akin to that reported in Moeller et al. (Moeller, Grohn et al. 2021). Thus, to rule out the possibility that such individual differences emanate from decision noise we next examined if pro-variance biases related to individual differences in anxiety or depression traits (Hong and Cheung 2015), including rumination, trait anxiety, intolerance of uncertainty, dysfunctional attitudes and self-report depression (see Methods for more details).

### Pro-variance biases and individual differences in anxiety and depression traits

In the discovery sample, we found that a general pro-variance bias (mean across the four same-mean blocks) was negatively correlated with rumination scores (Figure 2e, r(77) = –0.230, p = .041) as measured by the rumination response scale (Treynor, Gonzalez et al. 2003), a common trait in depression and anxiety (McLaughlin and Nolen-Hoeksema 2011). This suggests people with a higher rumination propensity were more risk averse. Further analyses, segregating different blocks, showed this effect was driven mainly by responses within unimodal distribution blocks (r(77) = 0.252, p = .025), and was not evident in bimodal blocks (r(77) = –0.079, p = .486). Moreover, the both-high mean and both-low mean blocks showed a similar pattern of negative correlation with rumination scores, but these did not reach formal significance level (r(77) = –0.175, p = 0.125 for the BHNH block, r(77) = –0.209, p = 0.065 for the BLNL block). This suggests individual propensities towards a pro-variance bias/risk-seeking is consistent across the two different contexts. However, we caution that it is important to validate these findings because the tasks (learning and making choices between options that are close in value) are subject to noise.

To validate an association between rumination and pro-variance bias, we conducted an independent replication experiment using the same design. Here we again found a negative correlation between pro-variance biases and rumination scores (Figure 2f, r(89) = –0.231, p = .027), again driven more by unimodal distribution (r(89) = –0.260, p = .013) as opposed to bimodal blocks (r(89) = –0.025, p = .817). As previously, the BHNH block and the BLNL block showed the same negative correlations with rumination scores, but this only reached trend-level significance (r(89) = –0.207, p = .049, and r(81) = –0.180, p = .088, respectively). As there was only a single testing block of the BHNH and BLNL block in each sample, the pro-variance bias calculated using a single block might have a low signal-to-noise ratio. So when we combined data from both samples, we found significant negative correlations between rumination scores and a preference for choosing the broader options in both unimodal blocks (r(168) = –0.153, p = .046 for the BHNH block (see Figure S2a), r(168) = –0.186, p = .015 for the BLNL block (see Figure S2b)).

### Computational basis for a pro-variance bias

**Figure 3:**
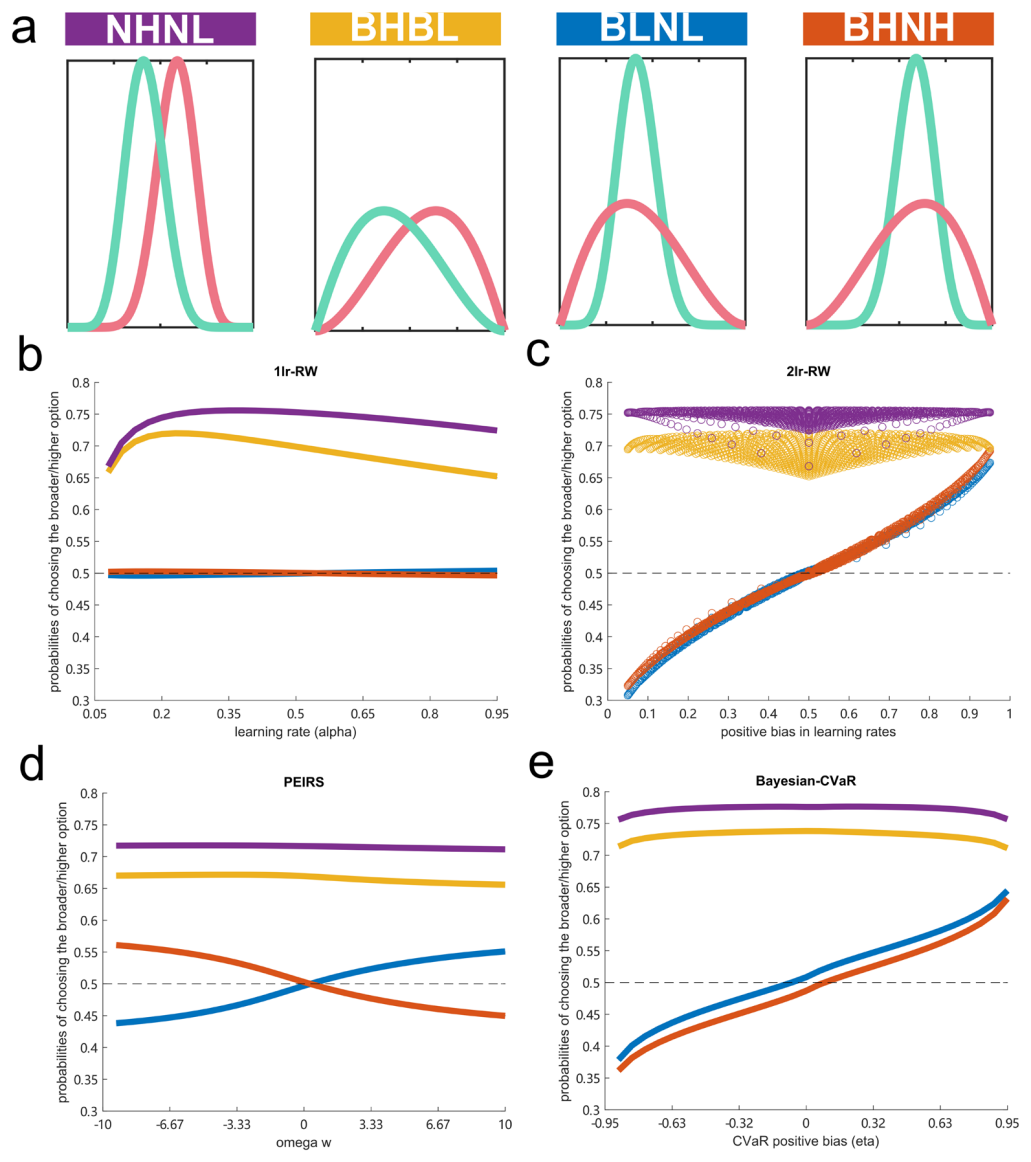
**Simulation results. a)**. The probability density function of the distributions for the 4 different conditions in the simulations. **b-e) simulation results for the 1lr-RW, 2lr-RW, PEIRS and the Bayesian-CVaR models respectively**. X-axes represent the learning rate from the 1lr-RW model, positive bias in learning rates from the 2lr-RW model, omega from the PEIRS model, and the CVaR term from the Bayesian-CVaR model, respectively. Y-axes represent the percentages of choosing the higher distribution for the different-mean conditions or the broader distribution for the same-mean conditions. Purple represents the both-narrow condition. Yellow represents the both-broad condition. Blue represents both the both-high condition. Red represents the both-low condition.

To better understand the computational mechanisms underlying pro-variance biases we conducted simulations using different models. To ground these models in our behavioural results, we set the simulations to match several benchmarks: 1) when two options presented in a given block have different means, the simulated agents should choose the higher value option significantly more often, with a lower performance if both options had broader distributions (both-broad) compared to narrow distributions (both-narrow); 2) when two options have the same means, the simulated agent should choose the broader distribution more when the means of the two options are both higher than a prior expectation, i.e., 7 for poker (both-high), but choose the narrower distribution more if the means of the two options are both lower than 7 (both-low); 3) the simulated agent should show a similar trend towards a pro-variance bias in the both-high and both-low conditions. Based on these considerations, we ran simulations across four different blocks/conditions: 1) both-narrow: the two options have both narrow distributions but different means (see Figure 3a NHNL); 2) both-broad: the two options have both broad distributions but different means (see Figure 3a BHBL); 3) both-low: the two options have the same means (both lower than 7) but different variances (see Figure 3a BLNL); 4) both-high: the two options have the same means (both higher than the prior, which is 7 in this study) but different variances (see Figure 3a BHNH).

We first simulated a simple Rescorla-Wagner(1lr-RW) agent, with two free parameters: 1) a learning rate (α) governing the learning process that controls the extent to which an agent incorporates trial-by-trial feedback into updating expected values and 2) an inverse temperature (β) for the decision-making process, that controls overall stochasticity in value-based choices (see Methods for more details). Because the decision-making process (i.e., the SoftMax function controlled by β) is a component shared across all our models, our prime focus for this simulation is on how the parameters in the learning process impact choice probabilities.

As expected, the simulated 1lr-RW agent learned the different-mean blocks well (Figure 3b), performing slightly better for the both-narrow (the purple line) compared to the both-broad (the yellow line) condition. However, the agent did not distinguish between the both-high (the blue line) and both-low conditions (the red line), nor did the learning rate render the agent more or less pro-variance biased (the probabilities of choosing the broader option stay at about 50%, i.e. chance level) for either of the same-mean conditions (Figure 3b). Because 1lr-RW agents always learn the mean of a distribution, it does not manifest any preference when two options have different variances but similar overall means.

To allow the simulated agents to form expected values, other than the means of the sequences, we enabled an RW agent to have different learning rates for positive and negative prediction errors (2lr-RW model). An agent with positive biased learning rates (positive learning rate > negative learning rate) will integrate more positive prediction errors into its expected values, forming an expected value higher than the actual mean. Such an agent should form an even more positively biased expected value when learning a broader distribution than a narrow one. Therefore, a positive agent should manifest a pro-variance bias, and vice versa for an agent with negatively biased learning rates, where a relative positivity in learning rates controls the expectile of a distribution the agent eventually learns (Lowet, Zheng et al. 2020).

Consequently, we simulated a 2lr-RW model using two free learning parameters: a positive learning rate for updating positive prediction errors and a negative learning rate for negative prediction errors, while the decision-making process is the same as for the 1lr-RW model. Positive learning rate biases were defined by the ratio of positive learning rates to overall learning rates (see Methods for more details). The simulation results showed a near-linear relationship between the percentages of choosing the broader options in the same-mean conditions and positive learning rate biases (Figure 3c). This is consistent with the hypothesis that risk aversion could result from negative learning. However, this model failed to capture the observed behavioural differences between the both-high and both-low conditions.

We next simulated the PEIRS model from Moeller et al. (Moeller, Grohn et al. 2021), designed to explain a difference in pro-variance bias between the both-high and both-low conditions. Essentially, this model learns both the mean (with a learning rate for updating value) and variance of a sequence (with a learning rate for updating the average level of surprises, i.e., absolute values of the prediction errors). Another term, omega, together with stimuli prediction errors of presented options (the both-high condition would have an overall positive stimuli error, and the both-low would have an overall negative prediction stimuli error), controls how the estimated variance is added to, or subtracted from, the learned expected values when making choices (see Methods for more details). For example, an agent with a positive omega should be more pro-variance biased in the both-high condition but, at the same time, prefer the narrow option in the both-low condition and vice versa for an agent with a negative omega (as shown in simulation results in Figure 3d). If people, in general, express a positive omega, this model should explain a relatively higher pro-variance bias in the both-high condition compared to the both-low condition, as shown in our empirical studies. However, in this model, pro-variance bias in the both-high condition should negatively correlate with pro-variance bias in the both-low condition. But, as we found consistent negative correlations with rumination scales in both-high and both-low conditions across both our samples, this model failed our 3rd benchmark criterion, as set out above.

**Figure 4:**
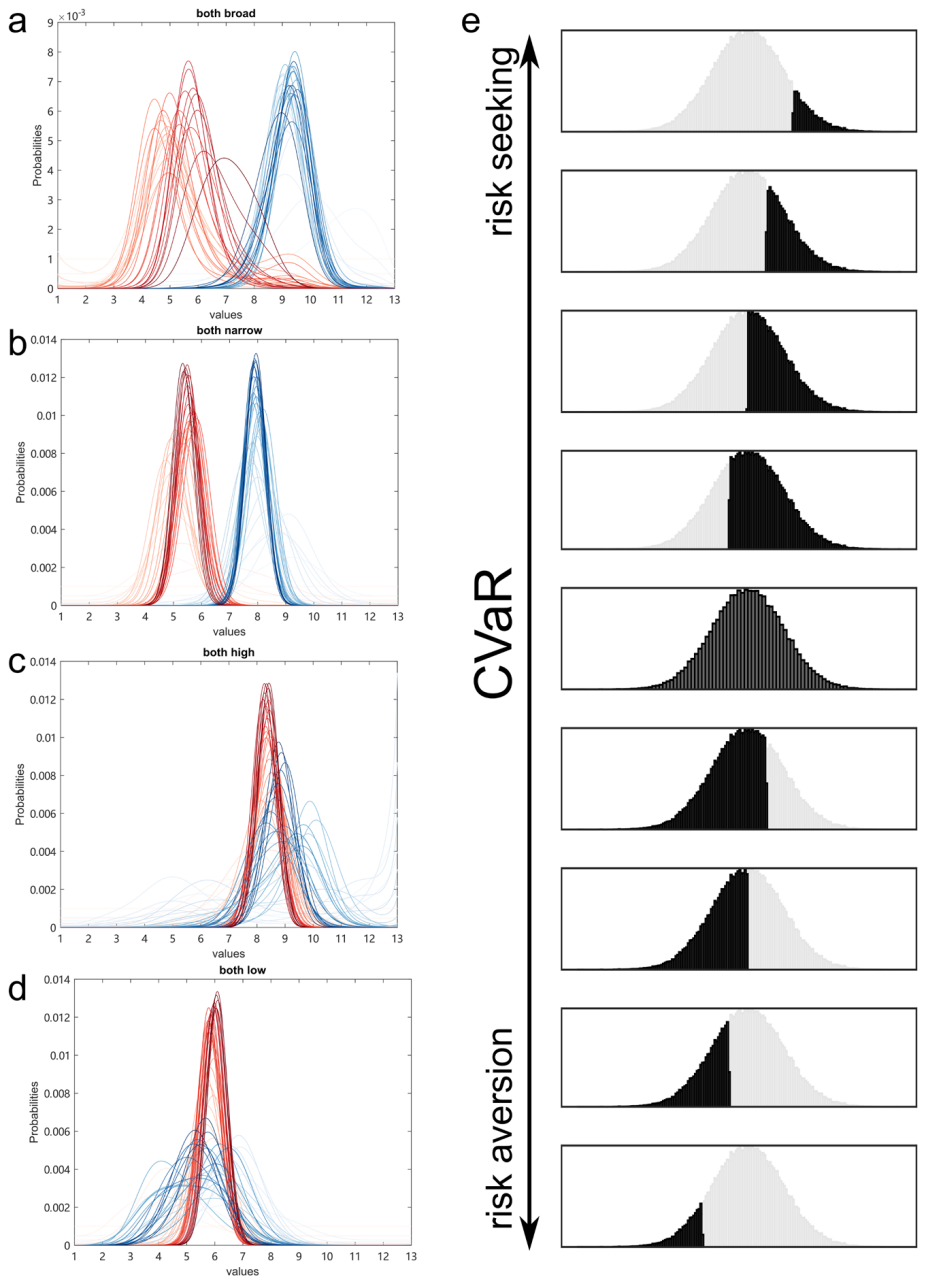
**The Bayesian-CVaR model and simulations. a–d)** Bayesian updating of the two card desks across 4 example blocks (a: BHBL, b: NHNL, c: BHNH, d: BLNL). The blue represents the higher or broader distributions. The red represents the lower or narrower distributions. Darker colours indicate more recently updated value distributions. **e)** the CVaR term controls which part of the distribution is read out for decision-making. The higher the CVaR level, the more risk-seeking/positive-bias an agent is. The black indicates which portion of the distribution is read out by the exemplar CVaR levels.

Finally, we implemented a newly described model, a Bayesian-CVaR model, which learns a probability density function of the possible values using Bayes’ rule (Figure 4a–d), and then applies a CVaR term (η), which reads out the expectations of either the lower part (η <0) or upper part (η > 0) of a distribution (Figure 4e), thereby controlling the overall risk level of an agent (see Methods for more details). The simulation results using this model met all three benchmarks: 1) the simulated agent performing well across the different-mean blocks; 2) The CVaR term controls a general propensity of pro-variance bias in both the same-mean conditions, consistent with a hypothesis that risk aversion could result from a negative interpretation bias; 3)At the same time, the agent showed consistently higher pro-variance biases in the both-high condition than in the both-low condition (Figure 3e).

To sum, both negative bias models, the negative learning model (2lr-RW model) and the negative interpretation model (the Bayesian-CVaR model), generated different propensities in terms of pro-variance biases in our simulations. However, the negative interpretation model (the Bayesian-CVaR model) best captured the behavioural characteristics observed in our empirical data.

### Model fitting and model comparisons

**Figure 5:**
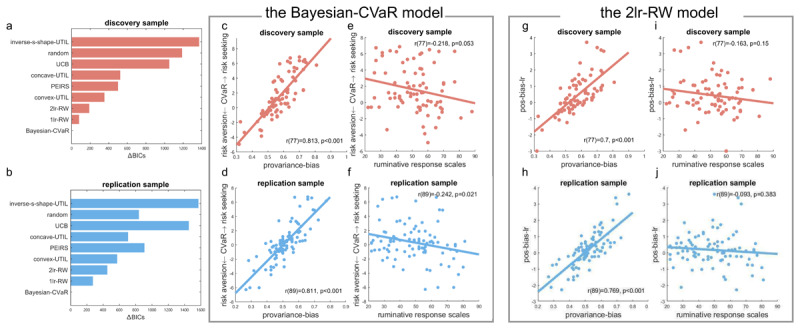
**Model estimates. a–b)** relative BICs for discovery and replication samples, respectively. **c–d)** correlations between the mean pro-variance bias and the CVaR estimates from the Bayesian-CVaR model for discovery and replication samples. **e–f)** correlations between the CVaR estimates and rumination response scales for discovery and replication samples. **g–h)** correlations between the positive bias in learning rates from the 2lr-RW model and mean pro-variance bias for the discovery and replication samples **i–j)** correlation between the positive bias in learning rates and rumination response scales for discovery and replication samples.

Next, we fitted simulated models to the empirical data. For completeness, we also included the utility models from Moeller et al (Moeller, Grohn et al. 2021) and the Upper Confidence Bound (UCB) model (Auer 2002)(see supplement). We found across both independent samples that the Bayesian-CVaR model fitted better than all other models, based on the Bayesian information criterion (BIC) (Figure 5a&b) and the exceedance probabilities (Fig S6). According to the simulation results above, we expected both the CVaR term from the Bayesian-CVaR model, and the positive bias in learning rates calculated from the 2lr-RW model, to capture individual differences in pro-variance bias in general. Indeed, we found that the CVaR estimates correlated with the pro-variance bias, showing the highest correlations across both samples compared to other models (Figure 5c&d, r(77) = 0.813, p < .001 for the discovery sample, and r(89) = 0.811, p < .001 for the replication sample). The positive bias in learning rates from the 2lr-RW model also showed strong correlations with pro-variance bias (Figure 5g&h, r(77) = 0.700, p < .001 for the discovery sample, and r(89) = 0.769, p < .001 for the replication sample). However, other models failed to capture the pro-variance bias: for the learning rate (α) estimates from the 1lr-RW model (discovery sample: r(77) = –0.279, p = .013; replication sample: r(89) = –0.125, p = .240), for the omega estimates from the PEIRS model (discovery sample: r(77) = –0.079, p = .491; replication sample: r(89) = –0.061, p = .567).

We next asked whether model estimates correlated with rumination response scales. We found that the CVaR term alone, from the Bayesian-CVaR model, correlated with rumination scale scores (Figure 5e&f, r(77) = –0.218, p = .053 for the discovery sample, and r(89) = 0.242, p = .021 for the replication sample). In contrast, key estimates from other models did not show significant correlations with rumination scores. For the positive bias in learning rates from the 2lr-RW model (Figure 5i&j, discovery sample: r(77) = –0.163, p = .150; replication sample: r(89) = –0.093, p = .383), the learning rate (α) estimates from the 1lr-RW model (discovery sample: r(77) = –0.130, p = .254; replication sample: r(89) = –0.011, p = .9204); for the omega estimates from the PEIRS model (discovery sample: r(77) = 0.144, p = .206; replication sample: r(89) = –0.067, p = .531).

## Discussion

Using a newly designed task, we provide evidence that participants’ choices under uncertainty manifest an enhanced pro-variance bias in good environments (when both options are better than expected) compared to choices in less good ones (when both options are worse than expected). Regardless of the environment, a pro-variance bias correlated negatively with participants’ rumination scores, indicating people who score high on this trait are less pro-variance biased or more risk averse in general. Based on these findings, we propose a new model, i.e., a Bayesian-CVaR model, that captures individual differences in pro-variance risk-seeking. In simulations, we show this model generates a range of risk-seeking and risk-averse behaviours. Modelling fitting and simulations indicated that a Bayesian-CVaR model best accounted for subjects’ behaviours, outperforming other models in both a discovery and replication study. Across both studies the risk-sensitive parameter CVaR correlated with individual differences in pro-variance bias and rumination scores.

In model-free analyses, we replicated an enhanced pro-variance bias when choice options were higher than the overall mean, as reported previously by Moeller et al. (Moeller, Grohn et al. 2021), an effect that depends on the establishment of the overall mean. Our task took advantage of the fact that, for poker playing cards, the range and mean are generally known to participants. Furthermore, a blocked-based presentation (resulting in less working memory demand) with full feedback (requiring fewer trials to learn and eliminate uncertainty-directed exploration) rendered the task shorter, easier, and more intuitive for online participants. This task design difference is one possible reason why the PEIRS model, which calculates an overall expectation, did not perform as well in this task version. Nevertheless, it is striking that for both Moeller et al.’s study and ours, although very different in design, both show a similar option mean modulation of pro-variance bias.

When we investigated individual differences in pro-variances bias, we found significant negative correlations, across both samples, between the pro-variance bias and rumination a common trait in anxiety and depression (McLaughlin and Nolen-Hoeksema 2011). However, we did not find clear evidence for an effect of means on the association between the pro-variance bias and rumination. Indeed, a similar pattern of negative association in the both-high and both-low blocks suggests that individual differences in risk sensitivity are more trait-like than context-dependent. Furthermore, our results indicate that being in a less favourable environment (available options having lower means) can increase the expression of risk aversion over time, as was also shown in Moeller et al. (Moeller, Grohn et al. 2021). One implication is that if people, for example, those with higher depression and anxiety traits, believe that rewards in their environment are scarce, then this might contribute to an explanation for their risk aversion propensity.

Our simulations and empirical findings were best accounted for by a Bayesian-CVaR model. Here an agent uses Bayes’ rule to update its beliefs regarding value distribution and then uses CVaR, a risk-sensitive term, to control how the learned distribution is utilized during decision-making. Combining Bayesian learning and CVaR has previously been implemented in Finance (Bodnar, Lindholm et al. 2022) and Artificial intelligence (Rigter, Lacerda et al. 2021, Lin, Ren et al. 2022). It is of interest that a theoretical paper that proposed using CVaR in modelling risk-sensitivity behavior in sequential tasks suggested a likely association with rumination (Gagne and Dayan 2022). Albeit using a much-simplified task, to the best of our knowledge, ours is the first study testing this class of model in human empirical data. Importantly, we show that CVaR estimates best capture individual differences in pro-variance bias and correlate with task-independent individual differences in trait rumination.

The Bayesian-CVaR model, combining a risk-sensitive term with a distributional representation, has two principal advantages. First, the CVaR term allows for greater flexibility in dealing with uncertainties. Indeed, the CVaR term in Bayes-adaptive Markov decision processes (MDPs) outperforms other models in stochastic environments (Rigter, Lacerda et al. 2021). Second, the Bayesian learner enables a richer representation of the distributions than the expected value. The advantage of distributional coding is evident in an observation that artificial agents with such coding outperform single expected value tracking agents (Dabney, Rowland et al. 2018).

A notable finding was a boosting of a pro-variance bias in extremely high variance (bimodal blocks) environments, which we speculate reflects the adoption of more varied learning strategies when values were bimodal. For example, participants might treat this context more like a probability learning task, i.e., treating big numbers (as winning) and small numbers (as losing) as two states with different probabilities. At the same time, correlations between pro-variance bias and rumination scores were primarily driven by unimodal blocks than the bimodal block, a finding consistent across the two samples. We note the bimodal block in our study has some resemblance to the Iowa gambling task (IGT) (Bechara, Damasio et al. 1994), where value distributions are also very bimodal, leading to winning a frequent small amount and occasional loss of a large amount. Indeed, studies using IGT find different behaviour patterns in people with depression compared to controls, however IGT behaviour correlation with depression scores is not well replicated (Buelow and Suhr 2009; Siqueira, Flaks et al. 2018; Arias, Patrick et al. 2022). Based on our findings, we surmise that unimodal distribution learning would show more consistent results in measuring individual pro-variance bias/risk-sensitivity, despite a limitation that for each participant we only have one each of the two unimodal blocks, one for the both-high block and one for the both-low block.

In summary, using a newly developed distribution learning task, we show that a Bayesian-CVaR model can generate risk-sensitive behaviour, as observed in our empirical data and capture individual differences in pro-variance bias. We suggest the task and the model provide a framework to study individual differences under uncertainty, offering a tool to further probe a disposition to depression and anxiety.

## Methods

### Participants and procedures

For our first study, we recruited 107 participants from the general population via Prolific for the discovery sample. Of these, 18 were excluded from the analyses because their mean accuracies for the different-means blocks were less than 60%; another 10 were excluded because they chose the same option for all the trials in at least one of the four same-mean blocks. Therefore, this left us with a final sample of 79 (38 females) participants aged 38.00 (±11.50).

For our replication study, we recruited 117 participants via Prolific. Of these, 21 were excluded from the analyses because their mean accuracies for the different-means blocks were less than 60%, 5 were excluded because they chose the same option for all the trials in at least one of the four same-mean blocks. The final replication sample consists of 91 (22 females) participants aged 30.72 (±10.15).

Participants were directed to pavlovia.org for the testing. The experiment was implemented using the software PsychoPy (v2021.1.4) (Peirce, Gray et al.). All participants also completed 5 questionnaires measuring different cognitive traits associated with anxiety and depression (Hong and Cheung 2015): 1) the rumination response scale (RRS) (Treynor, Gonzalez et al.). The RRS consists of 22 items each with a scale of 1(almost never) to 4 (almost always) from which participants choose. All item scores were summed to obtain a total RRS score. 2) the trait anxiety component from the state-trait anxiety inventory (tSTAI) (Spielberger 1983); 3) the Zung self-rating depression scale (SDS) (Zung 1965); 4) the intolerance of uncertainty scale (IUS) (Buhr and Dugas 2002); 5) the 17-item Dysfunctional Attitude Scale form A (DAS-A) (De Graaf, Roelofs et al. 2009) for the discovery sample, and a brief version of the Hypomanic Attitudes and Positive Predictions Inventory (Brief-HAPPI) (Mansell and Jones 2006) for the replication sample. The questionnaires were added as part of our online testing (after the main experiment) using a webpage-based tool (Morys-Carter 2021).

All participants provided informed consent before starting the task and were reimbursed a base rate and a bonus they earned during the learning task. This study was approved by the UCL Research Ethics Committee (16639/001) and is in accordance with the Declaration of Helsinki (Ashcroft 2008).

### Experimental design

Participants performed the magnitude learning task described in the Introduction section (Figure 1a). The underlying value distributions for each option differ in mean (high vs low) and variance (broad vs narrow). The mean of the high distributions is 8 (higher than the overall mean of 7) and 6 for the low distributions (see supplementary method for detailed descriptions). The task was composed of eight block types (Figure 1b): four different-mean blocks: broad-high vs broad-low (BHBL), narrow-high vs narrow-low (NHNL), broad-high vs narrow-low (BHNL), and narrow-high vs broad-low (NHBL); and four same-mean blocks: broad-high vs narrow-high (BHNH), broad-low vs narrow-low (BLNL), bimodal-high vs narrow-high (BiHNH), and bimodal-low vs narrow-low (BiLNL). Participants completed one block of each block type with each block containing 30 trials. The block order was randomized across participants.

At the beginning of every trial, participants had to choose between two card desks, cued by the different colours and/or patterns of the back of the poker cards. The same two card desks were shown throughout the block. Two new card desks (different colours and/or patterns from the ones shown before) were assigned to each new block. The positions (left or right side) of the two card desks were randomized from trial to trial. Participants made their choices by clicking on the cards. Participants were not limited in time to respond. One to two seconds after they made the choices, the card values drawn from each distribution would be shown simultaneously for three seconds, followed by 1–2 seconds of inter-trial intervals (ITI). If the chosen card number were higher than the unchosen card number, then participants would gain the points that were equal to the value differences between the two numbers for that trial; if the chosen card number were lower than the unchosen one, participants would lose the points that equal to the value differences between the two cards. The points wouldn’t change for that trial if the two card numbers were equal. Participants were told their won points would be added to their final reimbursement to encourage better performances.

### Statistical analyses

The percentages of choosing option a (the higher or broader option) were calculated for each block. The mean pro-variance biases were calculated by averaging the percentages of choosing the broader options for the 4 same-mean blocks, i.e., the BHNH block, the BLNL block, the BiHNH block, and the BiLNL block for each participant.

The statistical analyses were performed in IBM SPSS Statistics, version 26.0.0.0®. Using one-sample T-tests, we first examined whether participants’ preference for the higher option, for the different-mean block, or the broader option in the same-mean block, exceeded chance levels (50%) for each block. Then we ran a 2 × 2 repeated ANOVA analysis for the 4 same-mean blocks. The two factors were 1) overall means: high, i.e., the BHNH block and the BiHNH block, vs. low, i.e., the BLNL block and the BiLNL block; 2) variances of the options A: broad, i.e., the BHNH block and the BLNL block, vs. bimodal, i.e., the BiHNH block and the BiLNL block. Post hoc analyses were conducted using Bonferroni confidence interval adjustment. All correlation analyses in this paper were performed using Pearson’s Correlation two-tailed.

### Computational analyses

All model simulations and model fittings were performed using Matlab R2020b. Expect for the Bayesian-CVaR model, the other models used in this paper are all variants of the RW model (Rescorla 1971) which share key features and terms. In these models, expected values V_t_ are updated trial by trial using a proportion (controlled by learning rates α) of prediction errors δ, which is the deviation of reward outcome R_t_ and expected values V_t_. The outcome values (i.e. 1–13) were rescaled to 0.01–0.99. The prior expected value was set to 0.5. All models used apply a standard softmax function for decision-making processes (Daw 2011). In a two-arm bandit choice task, the probabilities of choosing an option (Pa) are linked to the differences between the learned expected values for the two options (see Equation 1). Inverse temperature β controls how stochastic the association is. Prior for β is log(β) ~ –3 – 3 for all models.


Equation 1 \[
{P_a} = \frac{1}{{1 + {e^{ - \beta \cdot \left( {{V_a} - {V_b}} \right)}}}}
\]

### Models

#### 1lr-RW

In this model, there is only one free parameter during the learning process, i.e., α, which controls how much the prediction errors (δ) are updated into the expected values (see Equation 2). Prediction errors are the differences between the outcome and the expected value for a given option. logit(α) ~ –4.6 ~ 4.6.


Equation 2 \[
{V_{t + 1}} = {V_t} + \alpha \cdot \delta
\]

Equation 3 \[
\delta = {R_t} - {V_t}
\]

#### 2lr-RW

In this model, two free parameters α_+_ and α_–_ are used to control how much the positive and negative prediction errors are integrated into the expected values, respectively (Equation 4 & Equation 5). Positive bias in learning rates (LR_pos-bias_) is defined as the ratio of the positive learning rate to the sum of the two learning rates (Equation 6). logit(α_+_/α_–_) ~ –4.6 ~ 4.6.


Equation 4 \[
{\alpha _{(\delta)}} = \left\{ {\begin{array}{*{20}{l}}
{{\alpha _ + },\;{\bf{if}}\;\delta \geqslant 0}\\
{{\alpha _ - },\;{\bf{if}}\;\delta < 0}
\end{array}} \right.
\]

Equation 5 \[
{V_{t + 1}} = {V_t} + {\alpha _{(\delta )}} \cdot \delta
\]

Equation 6 \[
L{R_{Pos - bias}} = \frac{{{\alpha _ + }}}{{{\alpha _ + } + {\alpha _-}}}
\]

#### PEIRS

In the PEIRS model, the expected values V and expected spread S of the outcomes are learned simultaneously using Equation 7 and Equation 8, respectively. The estimated spread S_t_ is combined with the overall prediction errors of the options δ_option_ to determine the value V’ (see Equation 10) used for the decision process (Equation 11). δ^t^_option_ is the overall prediction of mean values of the two options offered compared to a global mean, which is 0.5 in this task (Equation 9). For example, for the both-high blocks, δ^t^_options_ would be overall positive, δ^t^_options_ for the both-low blocks, would be overall negative. ω in the Equation 10 controls the direction and how much the estimated spread would influence the decision-making process. logit(α_Q_/αs) ~ –4.6 – 4.6. ω ~ –10 – 10. log(S_0_) ~ –4.6 – 0.


Equation 7 \[
{V_{t + 1}} = {V_t} + {\alpha _Q} \cdot {\delta _{outcome}}
\]

Equation 8 \[
{S_{t + 1}} = {S_t} + {\alpha _s} \cdot \left( {|{\delta _{outcome}}|-{S_t}} \right)
\]

Equation 9 \[
\delta _{options}^t = \frac{{V_a^t + V_b^t}}{2}-0.5
\]

Equation 10 \[
V_{t}^{'} = V_{t} + tanh(\omega \cdot \delta_{options}^{t}) \cdot S_{t}
\]

Equation 11 \[
{P_a} = \frac{1}{{1 + {e^{-\beta \cdot \left( {V_a^{\mathrm{'}}-V_b^{\mathrm{'}}} \right)}}}}
\]

#### Bayesian-CVaR

In this model, a probability density function θ of the value distribution for each option is learned using Bayes’ rules. The posterior belief P(θ_t+1_) of the value distribution is updated trial by trial by the combination of the prior belief P(θ_t_) and probability density function of the evidence for that trial P(R_t_) using Equation 12. The initial belief P(θ_0_) is set as a flat distribution using a Beta distribution Beta (1,1), assuming the probability of seeing each number is equally likely at the beginning of a block. The probability density function of the evidence is a Beta distribution Beta (eventα_t_, eventβ_t_) with a mean of R_t_ and the same variance (denoted as updatevar), as shown in Equation 14 & Equation 15, respectively. This means a new belief is a combination of a prior belief and a probability density function centering the recently shown number with a variance controlled by updatevar (set as a hyperparameter to 0.009 for this study). Eventα_t_ and eventβ_t_ can be calculated using Equation 16 & Equation 17 respectively (these two equations were derived using the solve equation function on Equation 14 and Equation 15 in MATLAB).


Equation 12 \[
P\left( {{\theta _{t + 1}}} \right) \propto P\left( {{R_t}} \right) \cdot P\left( {{\theta _t}} \right)
\]

Equation 13 \[
P({R_t}) = Beta(event\ {\alpha _t},event\ {\beta _t})
\]

Equation 14 \[
{R_t} = \frac{{event\ {\alpha _t}}}{{event\ {\alpha _t} + event\ {\beta _t}}}
\]

Equation 15 \[
updatevar\; = \frac{{\;event{\alpha _t} \cdot {\mathrm{event}}{\beta _t}}}{{{{\left( {\;event{\alpha _t} + \;event{\beta _t}} \right)}^2} \cdot \left( {event{\alpha _t} + event{\beta _t} + 1} \right)}}
\]

Equation 16 \[
event\alpha t = -Rt \cdot (Rt2-Rt + updatevar)./updatevar
\]

Equation 17 \[
\begin{array}{*{20}{c}}
{event{\beta _t} = ({R_t} - updatevar + {R_t} \cdot updatevar + 2 \cdot R_t^2}\\
{ + R_t^3)./updatevar}
\end{array}
\]

Now that we have the trial-by-trial estimated value distributions (Z), we apply CVaR to read out values as input to the softmax decision-making function. CVaR can be used to read out a part of either the lower (Gagne and Dayan 2022) or the upper end (Rockafellar and Uryasev 2000) of a distribution. Reading out the lowest generates the lowest value while reading out the highest end gives the highest value. Here we set the CVaR levels η from –0.95 to 0.95, with –0.95 reading out 5% lower end of a distribution and 0.95 reading out the top 5% of a distribution, while 0 reading out the mean of the whole distribution (see Figure 4e). CVaR level η = 0 means the agent makes decisions based on the unbiased mean of the estimated distributions. Positive η means the agent makes decisions based on a positively biased readout of the estimated distribution, and therefore is risk-seeking. Vice versa for negative η being risk averse. \[
{\rm logit}\left({\frac{{\eta + 1}}{2}} \right)\sim - 7 - 7
\] .

To do this, we first calculated the cumulative distribution function (CDF) of a distribution (Z) (Equation 18) and then find the corresponding percentile of the distribution, denoted as Value at Risk (VaR) (Equation 19). The CVaR is derived as either the mean of the distribution below the VaR (if η ≤ 0) or the mean of the distribution higher than the VaR (if η > 0) (Equation 20). CVaRs for the two options were put into a softmax function (Equation 1) to estimate the probability of choosing option a.


Equation 18 \[
F(Z) = P(Z \leqslant z)
\]

Equation 19 \[
Va{R_{\mathrm{\eta}}}(Z) = \left\{{\begin{array}{*{20}{l}}
{{\mathrm{min}}\{Z\mid F(Z) \geqslant 1 + \eta \},\;{\bf{if}}\; - 1 < \eta \leqslant 0}\\
{{\mathrm{min}}\{Z\mid F(Z) \geqslant \eta \},\;{\bf{if}}\;0 < \eta < 1}
\end{array}} \right.
\]

Equation 20 \[
CVa{R_\eta}(Z) = \left\{{\begin{array}{*{20}{l}}
{E\left[{Z\mid Z \leqslant Va{R_\eta}(Z)} \right],\;{\bf{if}}\; - 1 < \eta \leqslant 0}\\
{E\left[{Z\mid Z \geqslant Va{R_\eta}(Z)} \right],\;{\bf{if}}\;0 < \eta < 1}
\end{array}} \right.
\]

### Model fitting

All models were fitted in MATLAB using a variational Bayes’ approach. Only behaviour data from the four same-mean blocks were used for the model fitting. Because the different-mean blocks were very simple, many participants always chose the higher ones in these blocks. All trials from the same-mean blocks were used in the modelling fitting. Bayesian information criterion (BIC) was calculated for each model using the best-fitted parameters for each participant (Equation 21 & Equation 22). L̂ denotes the maximized value of the likelihood function of the model M, x: the observed data, n: the number of observations, k: the number of free parameters in the model. BICs for a random model were calculated with each decision probability set as 0.5 and k = 0. BIC scores were summed across participants, with lower sum BIC indicating better model fit. Delta BIC (plotted in Figure 5a&b) for each model was calculated by subtracting the BIC score of the best-fitting model in each experiment.


Equation 21 \[
BIC = k{\mathrm{ln}}(n)-2{\mathrm{ln}}(\hat L)
\]

Equation 22 \[
\hat L = p(x\mid \hat \theta, M)
\]

### Model simulation analyses

We simulated 4 task conditions: both-high, both-low, both-broad and both-narrow. Five hundred blocks of 30 trials were generated for each task condition for the agents to learn. The same events were used in the simulations for each model. We sampled evenly from each free parameter space for each model. The simulation result for a particular parameter was averaged across all other free parameters in the same model.

## Additional File

The additional file for this article can be found as follows:

10.5334/cpsy.114.s1 Supplementary Material. Supplementary methods, results and references.
