# Correction: Decision-Making, Pro-variance Biases and Mood-Related Traits

**DOI:** 10.5334/cpsy.132

**Published:** 2025-01-09

**Authors:** Wanjun Lin, Raymond J. Dolan

**Affiliations:** 1 Max Planck University College London Centre for Computational Psychiatry and Ageing Research, University College London, London WC1B 5EH, United Kingdom; 2 Welcome Centre for Human Neuroimaging, University College London, London WC1N 3BG, United Kingdom

**Keywords:** Risk-seeking, Rumination, Uncertainty, Pro-variance bias, Bayesian, Conditional Value-at-Risk

## Abstract

This article details a correction to: Lin, W., & Dolan, R. J. (2024). Decision-Making, Pro-variance Biases and Mood-Related Traits. *Computational Psychiatry, 8*(1), 142–158. https://doi.org/10.5334/cpsy.114.

## Correction

The Funding Information of Lin & Dolan (2024) should have included the grant codes to specify the funding received by the authors.

The full Funding Information description should state the following:

“This work was carried out within the framework of the Max Planck UCL Centre for Computational Psychiatry and Ageing Research, supported by the Max Planck Society. RJD is also supported by a Wellcome Investigator Award (No. 098362/Z/12/Z). The Wellcome Centre for Human Neuroimaging is supported by core funding from the Wellcome Trust (Grant No. 203147/Z/16/Z [to RJD]).”

The metadata for corresponding author Wanjun Lin has been updated in this correction article to reflect their current email.
