# Supplementary material for: Decision-Making, Pro-variance Biases and Mood-Related Traits
Source: Comput Psychiatr. 2024 Aug 21;8(1):142–58. doi: 10.5334/cpsy.114 (PMC11342847; doi:10.5334/cpsy.114)
Supplement: Supplementary Material. — Supplementary methods, results and references. [file cpsy-8-1-114-s1.pdf]

Supplement

SUPPLEMENTARY METHODS

Other Models

Concave utility. Several subjective utility functions from (Moeller, Grohn, Manohar, & Bogacz, 2021), including concave, convex and inverse s-shape, were fitted. In this model, an exponential function, see Equation 1& Equation 2, was used to allow for the concave subjective utility of the values in this experiment with the following setting: m =0.5, σ=0.5, $K_{concave}$ >0. Then the subjective utility values instead of the real values were used in 1lr-RW computations (Equation 3).

$$z = \frac{r - m}{\sigma}$$ **Equation 1**

$$U = m + \sigma \cdot \frac{1 - exp(-k_{concave} \cdot z)}{k_{concave}}$$ **Equation 2**

$$\delta_t = U_t - V_t$$ **Equation 3**

Convex utility. This model is identical to the concave utility model but replaces $K_{concave}$ with K_{convex}, with K_{convex} <0.

Inverse s-shaped utility. We also modelled the inverse s-shaped utility function using a sign-preserving power function (see Equation 4 & Equation 5). m =0.5, σ=0.5, $K_{inverse\ s\text{-}shaped}$ > 1.

$$z = \frac{r - m}{\sigma}$$ **Equation 4**

$$U = m + \sigma \cdot sign(z) \cdot |z|^{k_{inverse\ s-shaped}}$$ **Equation 5**

The Upper Confidence Bound model. In this model, estimated uncertainty (S) works as a bonus to the expected value of each option respectively (controlled by

the parameter γ), which ended up biasing towards the option with a higher variance(Auer, 2002; Gershman, 2018) (Equation 6). The expected values (V) and uncertainty (S) are updated the same way as in the PEIRS model. log(γ) ~ -3 - 2.3.

$$P_a = \frac{1}{1 + e^{-\beta \cdot ((V_a + \gamma * S_a) - (V_b + \gamma * S_b))}} \quad \textbf{Equation 6}$$

Simulating group-level effects

To test whether the models capitulate the group-level differences in pro-variance bias between the both-high and both-low conditions in the empirical data, we calculated the probabilities of choosing the broader option for each trial using the best-fitted model parameter set from each model for each participant. The probabilities were averaged within each block for each participant. The differences in pro-variances bias between the both-high and both-low conditions were calculated by subtracting the means of the pro-variance bias in the two both-low blocks (the BLNL and the BiLNL block) from the means of the pro-variance bias in the two both-high blocks (the BHNH and the BiHNH block). The differences were compared to the empirical dataset using two-sample t-tests for each model.

Value distributions for each option type

we used beta distributions for option values because Poker card numbers are bounded between 1 and 13. These values are generated using 'betarnd' function in MATLAB using Equation 7. And then round to the nearest integer. The parameter A and B for beta functions is derived using Equation 8 & Equation 9 (same as equation 16 &17 in the main text). The parameter m is set to 7/12 for high distributions and 5/12 for low distributions. The parameter var is set to 0.04 for broad distributions and 0.01 for narrow distributions.

Based on the above setting, the A and B parameters for the first four value distributions used in the magnitude learning task in this study are: 1) broad-high (BH): A=2.9612, B=2.1152; 2) broad-low (BL): A=2.1152, B=2.9612;3) narrow-high (NH): A=13.5949, B=9.7106;4) narrow-low (NL): A=9.7106, B= 13.5949.

For the bimodal distribution, they are drawn from a very high (m=9/12) and a very low (m=3/12) with a very low variance (var=0.005). For the very high distribution A=27.3750, B=9.1250; for the very low distribution A=9.1250, B=27.3750. The bimodal-high (BiH) is 1/3 probability of the very low distribution 2/3 of the very high distribution; while the bimodal-low (BiL) is 2/3 probability of the very low distribution 1/3 of the very high distribution.

$$\mathrm{V=12*@betarnd(A,B)+1} \quad \textbf{Equation 7}$$

$$A=-\mathrm{m}\cdot(m^2-\mathrm{m}+\mathrm{var})./\mathrm{var} \quad \textbf{Equation 8}$$

$$\mathrm{B} = (\mathrm{m} - var + m \cdot var + 2 \cdot m^2 + m^3)./var \quad \textbf{Equation 9}$$

Model parameter recovery

We conducted a model parameter recovery analysis for the top 3 best fitted models, i.e., the Bayesian-CVaR model, 1lr-RW model and the 2lr-RW model. The feedback from all four same-mean blocks used in the empirical studies was used for this analysis. For each parameter in each model, we sampled 30 (31 for CVaR to include 0) values equally spaced in each of the parameter value ranges. For each value combination, we calculated probabilities of choosing the broader option for each trial and simulated 50 agent behaviours based on estimated probabilities. We then fit the simulated behaviour data to the same model to get the estimated parameters. We then averaged a given recovered parameter value across all other parameter spaces.

Model recovery

We conducted a model parameter recovery analysis for the four models of interest in the main manuscript, i.e., the Bayesian-CVaR model, 2lr-RW model, 1lr-RW model and the PEIRS model. The feedback from all four same-mean blocks used in the empirical studies was used for this analysis. For each parameter, we randomly 100 sample values within its range. We then simulate the probabilities of choosing the broader option given the parameters in one of the models. We then generated behaviour choices based on the estimated probabilities. The choices were fit to all four models. Exceeding probability was calculated by the frequency of the winning model for each simulation based on AIC scores (Equation 10). We used AIC scores because AIC scores penalize for the number of parameters to a lesser extent than BIC scores, so less favour the Bayesian-CVaR (the winning model) and the 1lr-RW model.

$$AIC = 2k - 2\ln(\hat{L})$$

Equation 10

Bayesian model selection with protected exceedance probability

We first calculated the unprotected exceedance probability (UEP), which is the probability that a given model is higher than any other model based on AIC scores. Protected exceedance probability (PEP) is derived using Equation 11, with a Bayesian Omnibus Risk (BOR) assuming all models (8 in total in this study) are equally possible.

$$PEP_i = \frac{UEP_i - BOR}{1 - BOR}$$

Equation 11

Model fitting to data from Moeller et al's

We fitted the four models of interest, i.e., Bayesian-CVaR model, 1lr-RW model, 2lr-RW model and PEIRS model, to Moeller's data (Moeller et al., 2021), where the overall mean could drift during learning. The model fitting process remains the

same for all other models, except allowing the mean to be determined dynamically by the expected values of all four options in the PEIRS model.

Additional behaviour data analysis

One of the differences between the task in the current study and the one from Meller et al's (Moeller et al., 2021) is that by using a poker framework, we assume the overall mean is mostly determined by rich daily life experiences that participants have with poker, therefore we assume the overall mean expectation in this task doesn't change. We designed the task so that the different-mean blocks have an overall mean of 7, therefore those blocks won't change the overall mean. Therefore, here we examine whether there is evidence that overall mean changes from a previous block (which can only happen for a same-mean block) impact the pro-variance bias in the following block. Because a block type cannot followed by itself, to avoid bias in pro-variance following a both-high or a both-low block, we calculated the probabilities of choosing the broader option in a BLNL and BHNH block following a BiHNH (both-high) or a BiLNL (both-low) block, and probabilities of choosing the broader option in a BiHNH and BiLNL following a BHNH(both-high) or a BLNL (both-low) block respectively. We then combine them to be closer to the pro-variance bias we calculated in the main manuscript. We combined the discovery and replication datasets for this analysis. We then ran an independent sample t-test.

SUPPLEMENTARY RESULTS

Demographic information

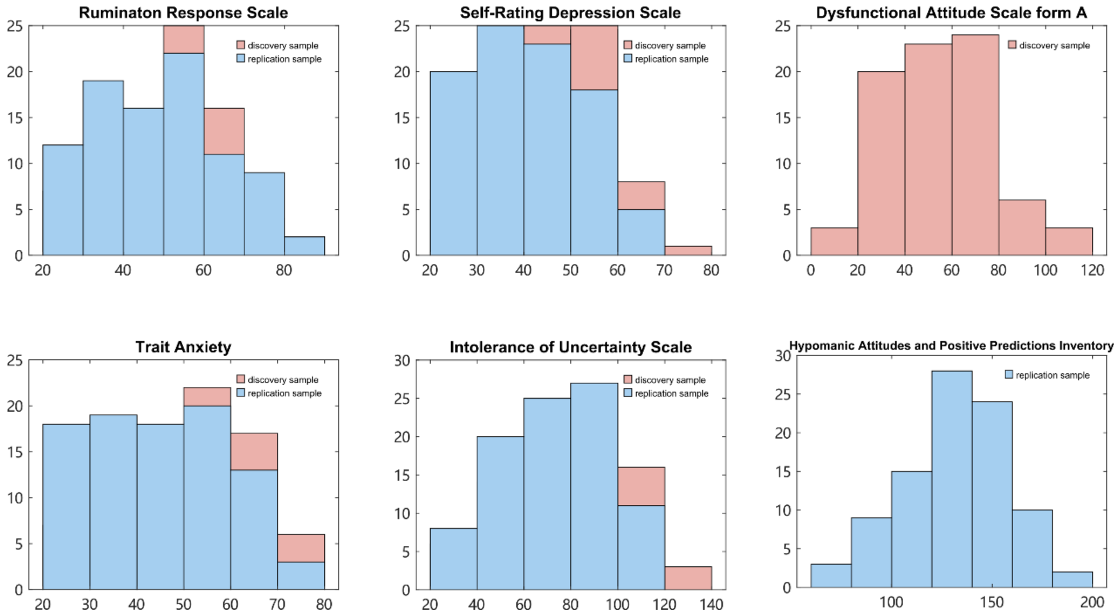

Fig.S 1 histograms of questionnaires scores.

Pro-variances bias and rumination across both samples

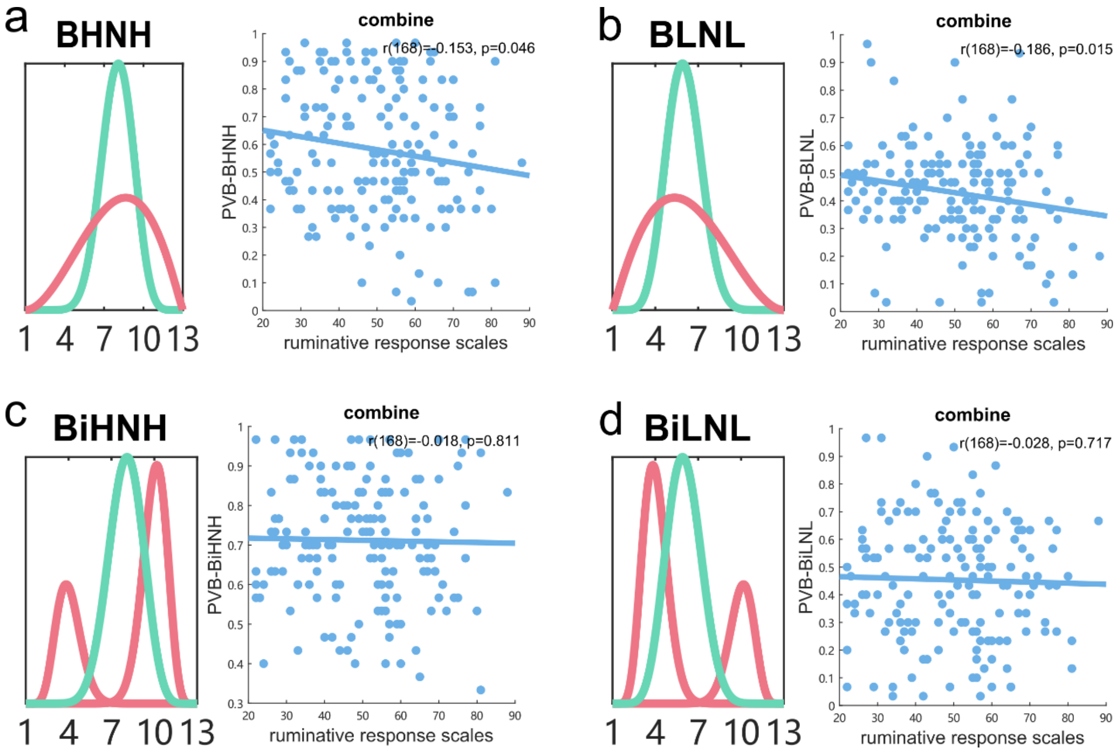

Fig.S 2 The procentages of choosing the broader option for the BHNH (a), the BLNL (b), the BiHNH(c) and the BiLNL (d) blocks respectively.

Correlations between pro-variance bias and other questionnaires

For completeness, in the replication sample, scores from a brief version of the Hypomanic Attitudes and Positive Predictions Inventory(Brief-HAPPI) scores, a measure for hypomanic traits (Mansell & Jones, 2006), were also negatively correlated with general pro-variance biases (r(89)=-.263, p=.012). However, all other questionnaire data (including the trait anxiety comportment from the state-trait anxiety inventory (tSTAI) (Spielberger, 1983), the Zung self-rating depression scale (SDS) (Zung, 1965), the intolerance of uncertainty scale (IUS) (Buhr & Dugas, 2002), and the 17-item Dysfunctional Attitude Scale form A (DAS-A) (De Graaf, Roelofs, & Huibers, 2009)) showed no significant correlation with the pro-variance biases in either of the samples (all p>.132).

Emergence of pro-variance biases over trails in a block

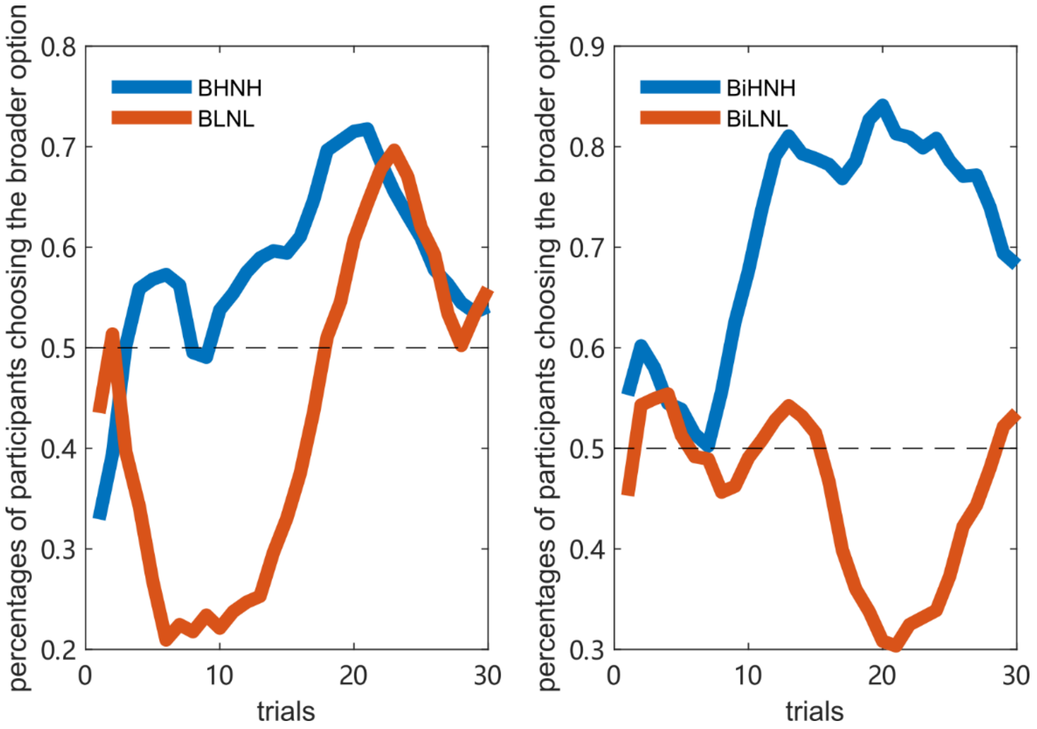

Fig.S 3 Percentages of participants (from both samples, n=170) choosing the broader option for the BHNH (left, blue) and BLNL (left, red) block, and the BiHNH (right, blue) and BiLNL(right, red) block for each trial.

Recapitulating group-level differences in pro-variance bias using simulations

To test whether the models could recapitulate group-level differences in pro-variance bias between the both-high and both-low mean conditions, as described above (see Fig2 a&b and also the last bars in Fig.S 4 a&b), we calculated the probabilities of choosing the broader option for the both-high (the BHNH and BiHNH block) and the both-low mean conditions using the best fitted parameter estimates from each model for each participant. We found that in both samples the Bayesian-CVaR produced the pro-variance bias differences between conditions closest to the empirical datasets ($t(156)=-1.541$, $p=.125$ for the discovery sample, $t(180)=-1.180$, $p=.239$ for the replication sample). The PEIRS model produced similar differences between the two conditions. But the simulated condition differences from the PEIRS model was significantly smaller than the differences observed in the empirical datasets ($t(156)=-3.043$, $p=.003$ for the discovery sample, $t(180)=-3.410$, $p<.001$ for the replication sample). The differences that the other models produced were all very significantly less than the empirical datasets (all $t(156)>-8.606$, $p<.001$ for the discovery sample, and all $t(180)>-7.737$, $p<.001$ for the replication sample).

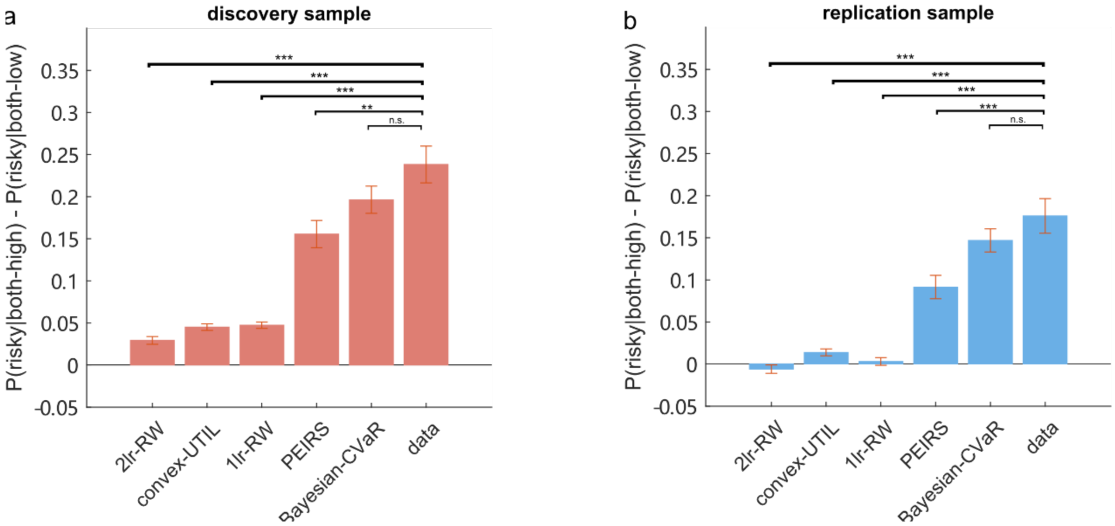

Fig.S 4 Difference in risk preferences between the both-high and both-low condition for simulated and empirical datasets for a) the discovery sample and b) the replication sample. Error bars indicate

standard errors (s.e.); *p < 0.05; **p < 0.01; ***p < 0.001.

Model robustness analysis results

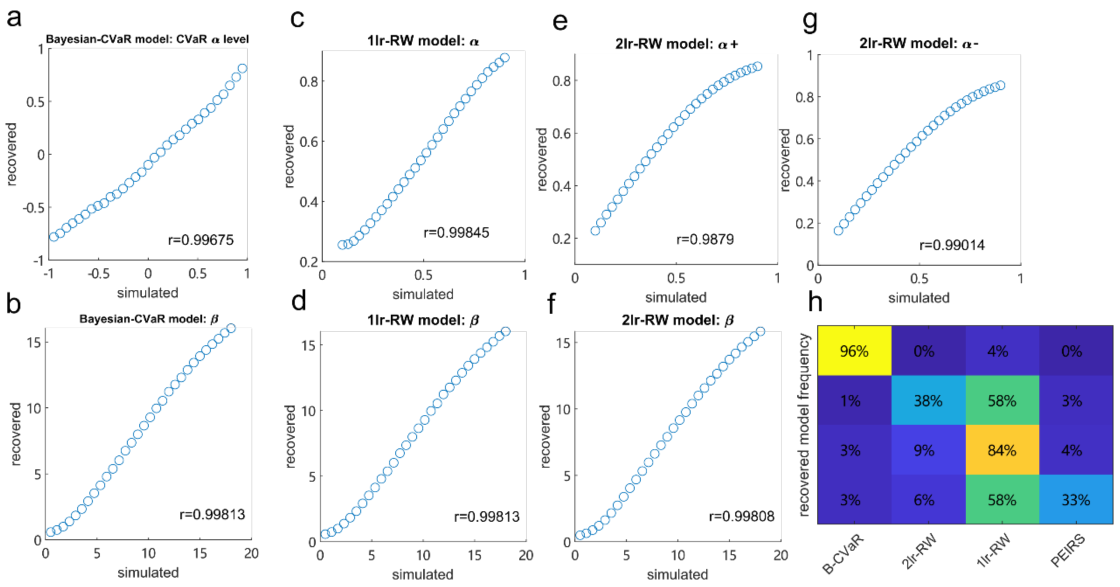

Fig.S 5 a-b) model parameter recovery of the Bayesian-CVaR model, for CVaR alpha level and beta parameters respectively. **c-d)** model parameter recovery of the 1lr-RW model, for learning rate and beta parameters respectively. **e-g)** model parameter recovery of the 2lr-RW model, for the positive and negative learning rates and beta respectively. **h)** model recovery confusion matrix including the Bayesian-CVaR model (B-CVaR), 2lr-RW model, 1lr-RW model, and PEIRS model.

Model comparisons using protected exceedance probabilities

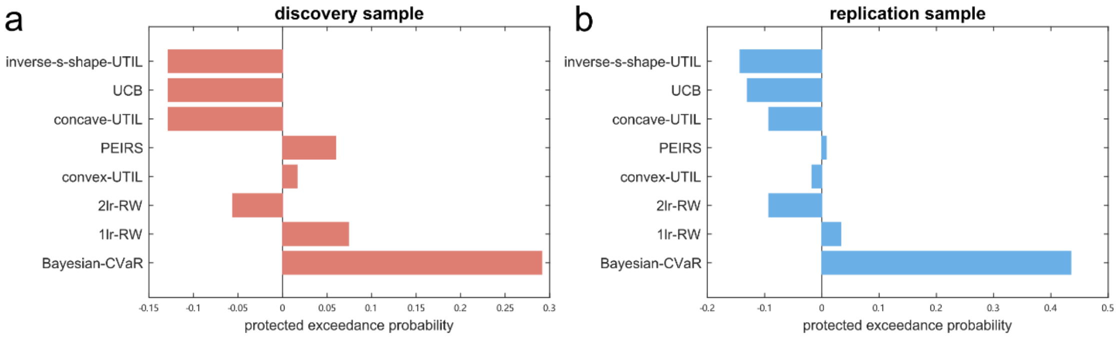

Fig.S 6 Protected exceedance probabilities for each model for the discovery (a) and replication (b) sample respectively

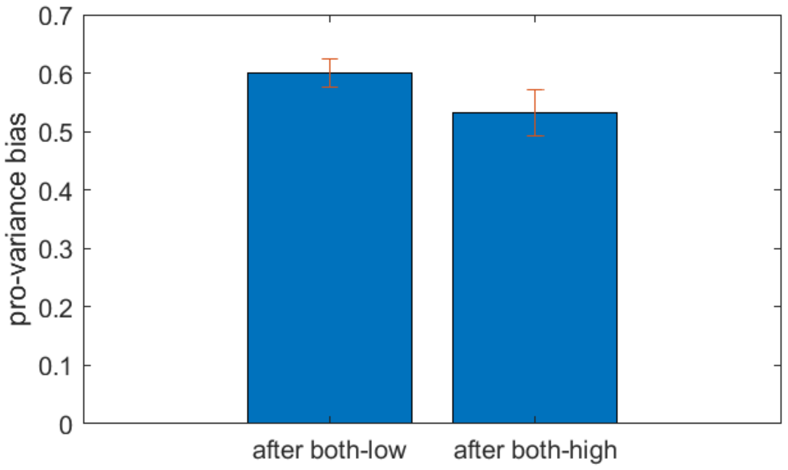

Fig.S 7 The probabilities of choosing the broader option (pro-variance bias) immediately after a both-low (BLNL or BiLNL) or a both-high (BHNH or BiHNH) block.

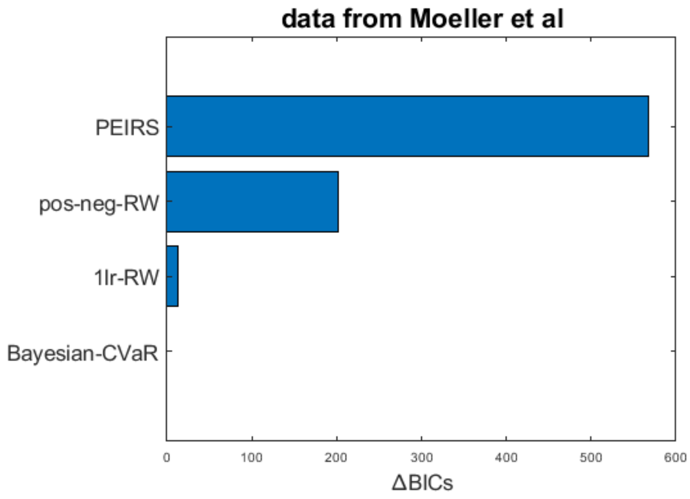

Fig.S 8 relative BICs for fitting the models to the data from Moeller et al(Moeller et al., 2021).

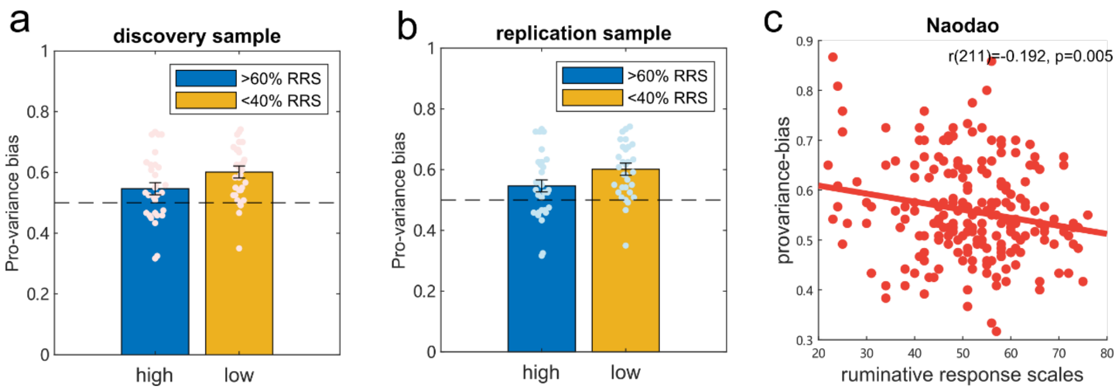

Fig.S 9 a-b) pro-variance bias for people with high (>60% of the sample) and low (<50% of the sample)

rumination scores for discovery (t(60)=-2.105, p=.040) and replication samples (t(67)=-2.260, p=.027) respectively. **c)** pro-variances bias negatively correlated with rumination scores in another independent online sample with a doubled sample size (n=213).

References

Auer, P. (2002). Using confidence bounds for exploitation-exploration trade-offs. *Journal of Machine Learning Research, 3*(Nov), 397-422.

Buhr, K., & Dugas, M. J. (2002). The intolerance of uncertainty scale: Psychometric properties of the English version. *Behaviour Research and Therapy, 40*(8), 931-945.

De Graaf, L. E., Roelofs, J., & Huibers, M. J. (2009). Measuring dysfunctional attitudes in the general population: The Dysfunctional Attitude Scale (form A) Revised. *Cognitive Therapy and Research, 33*, 345-355.

Gershman, S. J. (2018). Deconstructing the human algorithms for exploration. *Cognition, 173*, 34-42.

Mansell, W., & Jones, S. H. (2006). The Brief-HAPPI: A questionnaire to assess cognitions that distinguish between individuals with a diagnosis of bipolar disorder and non-clinical controls. *Journal of Affective Disorders, 93*(1-3), 29-34.

Moeller, M., Grohn, J., Manohar, S., & Bogacz, R. (2021). An association between prediction errors and risk-seeking: Theory and behavioral evidence. *PLOS Computational Biology, 17*(7), e1009213. doi:10.1371/journal.pcbi.1009213

Spielberger, C. D. (1983). State-trait anxiety inventory for adults.

Zung, W. W. (1965). A SELF-RATING DEPRESSION SCALE. *Arch Gen Psychiatry, 12*, 63-70.

doi:10.1001/archpsyc.1965.01720310065008
